# On the Treatment of Typhus and Typhoid Fevers

**Published:** 1852-12

**Authors:** 


### *On the Treatment of Typhus and Typhoid Fevers.* By Dr. Todd.

The general principles contained in this brief article will be found to accord in a striking manner with the views relating to the same subject submitted by us in former numbers of this Journal.—Editor Buffalo Med. Journal.

One important feature of fever, whether it be Typhus or Typhoid, whether diarrhœa be present or not, is *depression.* The disease is adynamic, and great attention must therefore be paid to supplying the patient with a proper nutriment. The basis of his diet should be proteinaceous matters, in such a state that the stomach shall have little or nothing to do to bring them to a condition fit for absorption. In the animal broths, well made, and in milk, you have food which answers to this description. The former, on the whole, are probably the best. Milk is less easily digested, and does not always harmonize with other matters necessary to be given. Farinaceous matters may be introduced also in small quantities. A great secret of success in administering support to patients under these circumstances is this—to give it very frequently in small quantities—quantities so small, that the whole or greater part of one supply may be absorbed before the next supply is brought; and also not to give a variety of food. Keep to milk and beef tea, or other broth, or to broth and farinaceous matter.

In the great majority of cases you must, I think, give stimulants, and give them early. They will often fail because begun too late. The best stimulants are brandy and port wine, with either of which chloric ether will go as well as a medical stimulant; any one of the three will often suffice alone. Port wine and brandy ought not to be given together, simply because in general the stomach does not digest well two kinds of stimulants. The same rule as to frequent administrations, and in small quantities, which I have already laid down for food, holds with equal if not greater force in giving stimulants.

In my opinion, the question in the treatment of fever is, not whether you shall give stimulants, but how much you shall give. In many you may give as much as half an ounce every half hour, or even an ounce of brandy, with advantage; but this is in bad cases. On this point you must be guided by the rapidity and compressibility of the pulse, and by the intensity of the heart's action. An important character of the pulse is found in the manner in which it strikes the finger; if vacillating, it is a decided indication for the use of stimulants. The strength of the heart's action, especially of the second sound, is also a good indication. If either sound be weak, but especially the second, you need not fear to give stimulants freely. An impulsive character of the heart's action with a feeble sound, also denotes the use of stimulants. Under such a plan of treatment, in which nutritious fluids and stimulants are given freely and from an early period, we find our mortality in fever to be small; we very seldom lose a case of fever. I do not allow myself to be deterred from giving stimulants by the state of the bowels; I know that many have a fear that much alcoholic stimulants irritates the bowels. If the alcohol be given in small quantities each time, it can not irritate it by direct contact, because it is absorbed before it reaches the intestines. Alcoholic stimulants, if not given too much at a time, are digested in the stomach, and the alcohol gets immediately absorbed and carried into the circulation. If it does harm, it does so from being in the blood; yet I must confess I have never seen satisfactory evidence of this.

We must also pay close attention to the bowels. If diarrhœa be present, it must be checked by those astringents which contain tannin; as the infusion or tincture of rhatany, catechu, of matico, of logwood, or you may give enemata with small quantities of laudanum. I find chalk often fails, and moreover it is liable to this objection, that as it does not dissolve, its particles may add to the irritation of the blood, by sticking in the ulcerated or inflamed patches. Counter-irritation over the abdomen by mustard, turpentine or blister, is also frequently of great use. If there is hemorrhage, you may give small doses of turpentine, five minims repeated every three or four hours, and in such cases, turpentine must be used as an external counter-irritant to the belly.

Another feature in these cases is, the frequent occurrence of bronchitis or bronchial congestion, indicated by rhonchus and crepitation. The bronchial congestion and diarrhœa are frequently the most difficult symptoms we have to deal with in those cases in which we find maculæ. The bronchitis may be relieved by the free application of turpentine stupes or blisters to different parts of the chest, at the same time or in succession; and though in such cases we must carefully watch the effects of our stimulants, we must not think of lowering our patient by bleeding, or by the application of any antiphlogistic remedies.—*Medical Gazette*, 1851.

